# HydroBound-ML: Automated Water-Surface-Mask Generation for Airborne Lidar Bathymetry Processing Using Hybrid Machine Learning and Object-Based Image Analysis

**DOI:** 10.3390/s26196065

**Published:** 2026-09-24

**Authors:** Patryk Wróblewski, Anna Fryśkowska-Skibniewska

**Affiliations:** Department of Imagery Intelligence, Faculty of Civil Engineering and Geodesy, Military University of Technology, 2 gen. Sylwestra Kaliskiego St., 00-908 Warsaw, Poland; anna.fryskowska@wat.edu.pl

**Keywords:** Airborne Lidar Bathymetry (ALB), machine learning, random forest, Object-Based Image Analysis (OBIA), water boundary delineation, topo-bathymetric scanning, exponential decomposition

## Abstract

**Highlights:**

**What are the main findings?**

- A novel hybrid framework (HydroBound-ML) was developed by decoupling Random Forest semantic classification from Object-Based Image Analysis (OBIA) geometric vectorization to delineate water boundaries.
- The proposed Coarse-to-Fine architecture effectively resolves spectral confusion between turbid water and urban gray infrastructure, achieving a Precision of 0.99 and an F1-score of 0.95.

**What are the implications of the main findings?**

- The automated generation of precise spatial masks prevents false bathymetric target extraction in Airborne Lidar Bathymetry (ALB) processing.
- Integrating Big Data operationalizations provides a highly scalable and precise alternative to manual masking for updating hydrographic geographic information systems (GISs).

**Abstract:**

Airborne Lidar Bathymetry (ALB) requires accurate water-surface masks for precise bathymetric target extraction from full-waveform data, such as via Exponential Decomposition, preventing false target extraction over adjacent land masses. HydroBound-ML is an image-based tool developed to automatically generate accurate water-surface masks for subsequent Airborne Lidar Bathymetry (ALB) processing. Accurate water-surface masks are essential for reliable bathymetric target extraction from full-waveform data, such as via Exponential Decomposition, as they prevent false target extraction over adjacent land areas. Since static topographic databases are frequently obsolete due to river dynamics and manual delineation is prohibitively time-consuming, this study automates the water boundary vectorization process. We introduce HydroBound-ML, an open-source, hybrid Geographic Information System (GIS) framework utilizing simultaneous high-resolution multi-spectral imagery. By decoupling semantic classification from geometric vectorization, a Random Forest classifier operates within a high-dimensional spatial–spectral feature space to identify core water masses. Precise physical boundaries are subsequently delineated using Simple Linear Iterative Clustering (SLIC) superpixels via Object-Based Image Analysis (OBIA). The system integrates advanced Big Data operationalizations, including Cloud Optimized GeoTIFFs (COGs) and hardware-accelerated Boolean topology editing. Quantitative benchmarking on complex, turbid river environments demonstrates that HydroBound-ML decisively resolves the spectral ambiguity between water and gray infrastructure. Achieving exceptional accuracy (Precision = 0.99, F1-Score = 0.95) and outperforming standard thresholding techniques (NDWI, OTSU), HydroBound-ML provides a highly precise, scalable alternative to manual masking for ALB processing.

## 1. Introduction

The accurate mapping of shallow water bathymetry is crucial for hydrological modeling, flood risk assessment, and fluvial morphology [1,2,3,4]. Airborne Lidar Bathymetry (ALB) utilizes active optical systems, typically emitting short green-wavelength laser pulses, to record returns from the water surface, water column, and seabed. Topo-bathymetric systems, such as the Riegl VQ-880-GII (RIEGL Laser Measurement Systems GmbH, Horn, Austria), leverage full-waveform processing to extract high-resolution topography across both terrestrial and aquatic domains [5,6,7]. Within commercial ALB processing environments like RiPROCESS (version 1.9.7.4 RIEGL Laser Measurement Systems GmbH, Horn, Austria), advanced algorithms such as Exponential Decomposition are employed to separate overlapping waveforms and extract submerged targets from the green laser channel [8,9,10,11].

A fundamental prerequisite for these extraction algorithms is the definition of a precise spatial boundary. This boundary acts as a constraining polygon equipped with ellipsoidal altitudes, explicitly restricting the bathymetric extraction process to physical water bodies. Inaccuracies in this boundary lead to severe extraction failures, where the algorithm attempts to locate artificial seabed targets beneath dry land. Given the high temporal dynamics of fluvial environments (e.g., meandering channels, ephemeral islands), static state databases (e.g., BDOT10k, BDOO in Poland) are frequently obsolete [12].

In this workflow, the water boundary is not extracted from the LiDAR point cloud itself. Instead, HydroBound-ML (version 1.0.7; available online: https://github.com/PWroblewskiWAT/HydroBound-ML, accessed on 21 September 2026) uses the native RGB and NIR imagery acquired simultaneously by the optical cameras integrated with the topo-bathymetric system. These images are used to generate the water-surface mask, which is then provided as a spatial selector for the subsequent ALB processing and bathymetric target extraction. In this way, the proposed method serves as an image-based preprocessing step within the ALB workflow rather than as a LiDAR point cloud classification method. The automated generation of precise spatial masks prevents false bathymetric target extraction in Airborne Lidar Bathymetry (ALB) processing.

While deep learning and standard machine learning (ML) approaches have been utilized for water classification [13,14,15,16,17], their application in high-resolution, complex fluvial environments often suffers from severe spectral confusion. Specifically, the “Information Ceiling” paradox occurs when turbid water, dark asphalt, and shadowed infrastructure share identical spectral signatures in the RGB and Near-Infrared (NIR) spectrums. Consequently, automated masks tend to erroneously encompass bridges or fail to delineate sharp shorelines. Indeed, the reliable spatial decoupling of water surfaces from adjacent gray infrastructure remains a notoriously persistent challenge in airborne LiDAR processing, driving continuous advancements in both traditional point cloud classification and novel deep learning architectures [18,19]. Although advanced deep learning (DL) architectures provide state-of-the-art semantic segmentation, their operational deployment in large-scale hydrographic campaigns is often constrained by the necessity for massive, densely annotated training datasets and prohibitive GPU memory requirements during the inference of Very High Resolution (VHR) datasets. Therefore, this study deliberately adopts a conventional machine learning approach (Random Forest). The RF algorithm was selected for its high interpretability, computational efficiency, and robust capability to handle the proposed high-dimensional spatial–spectral feature space without requiring specialized cluster infrastructure. Crucially, this lightweight architecture enables rapid, quasi-real-time model retraining within the proposed ‘Human-in-the-Loop’ framework.

The main aim of this study was to develop and evaluate an automated method for generating accurate water-surface masks to support the processing of large-scale Airborne Lidar Bathymetry (ALB) datasets. Specifically, the study aimed to combine spectral, textural, and geometric information within a machine-learning framework, generate a spatially precise water-surface selector from native RGB and NIR imagery acquired during ALB surveys, compare the proposed approach with established water-delineation methods, and evaluate its performance using a spatially independent test area. The resulting water-surface mask is intended to define the area in which subsequent ALB waveform processing and bathymetric target extraction are performed. The core methodological innovation of this research is the deliberate architectural decoupling of semantic classification from geometric vectorization to resolve the inherent “Pixel-based Texture Classification Edge Paradox”. By dynamically fusing contextual machine learning (for robust semantic understanding) with Object-Based Image Analysis (for precise physical gradient-snapping), the proposed framework achieves a level of boundary precision and infrastructural noise rejection that isolated machine learning classifiers or conventional thresholding simply cannot attain.

This framework shifts the paradigm from flat pixel-based thresholding to a multi-dimensional, Coarse-to-Fine architecture that fuses contextual machine learning with Object-Based Image Analysis (OBIA). Additionally, it addresses the massive computational overhead of ALB operations by implementing state-of-the-art spatial data engineering, enabling quasi real-time human-in-the-loop corrections over vast topographical datasets.

The contribution is not a new Random Forest or a new segmentation algorithm; rather, it is a Coarse-to-Fine integration of contextual semantic classification with object-based geometric refinement for generating accurate water-surface masks from native multispectral imagery acquired during ALB surveys for subsequent ALB processing.

In summary, HydroBound-ML provides an accurate, scalable, and reproducible framework for automatic water-boundary delineation in large airborne bathymetric LiDAR datasets.

## 2. Materials and Methods

This section details the data acquisition parameters and the computational architecture developed for the HydroBound-ML framework. First, the specific sensor configuration and flight mission parameters utilized to acquire the topo-bathymetric dataset are defined (Section 2.1). Subsequently, the methodology for extracting water boundaries is presented progressively, starting from the Tri-Index Spectral Fusion (Section 2.2) and the integration of a multi-scale contextual feature space for the machine learning model (Section 2.3). Finally, the framework explains the semantic–geometric decoupling employing Object-Based Image Analysis (Section 2.4) and the Big Data operationalization strategies implemented to process massive spatial datasets efficiently (Section 2.5). Finally, the hardware environment enabling this computational efficiency is outlined (Section 2.6), followed by a comprehensive description of the validation strategy and benchmark methods used to rigorously evaluate the algorithm’s performance (Section 2.7).

### 2.1. Sensor Configuration, Flight Parameters and Data Acquisition

The primary data was acquired utilizing the Riegl VQ-880-GII (RIEGL Laser Measurement Systems GmbH, Horn, Austria) topo-bathymetric system, structurally integrated with two PhaseOne iXM-RS150F (Phase One A/S, Frederiksberg, Denmark) photogrammetric cameras (capturing separate RGB and NIR spectra) (Figure 1). It is critical to clarify that the 2D optical imagery utilized in this framework is inherently native and captured simultaneously by these integrated PhaseOne cameras. The 2D orthomosaics are not derived from rasterizing the 3D LiDAR point cloud, thereby entirely avoiding any geometry-to-raster transformation accuracy loss. This multi-sensor integration allows the algorithm to natively exploit high-resolution spectral radiometry (RGB and NIR) that the raw green-wavelength LiDAR lacks prior to point classification. The role of these optical data is specifically to provide the spatial information required for water-surface-boundary delineation. HydroBound-ML processes these native RGB and NIR observations to generate a water-surface selector, which is subsequently used to constrain the spatial domain of the downstream ALB processing. The LiDAR measurements therefore constitute the operational context and intended downstream application of the generated mask, rather than the direct input to the image-classification stage.

The study area comprised a 20 km, morphologically complex stretch of the Pilica and Vistula rivers in the Mazovian Voivodeship, Poland (Figure 2).

A critical operational challenge in ALB missions is balancing the penetration capabilities of the green channel (532 nm) with stringent ocular safety regulations (Laser Standard 2014 [20]). To achieve maximum bathymetric depth, a high single-pulse energy at a lower Pulse Repetition Rate (PRR) is preferred. However, defaulting to such settings inherently extends the Extended Nominal Ocular Hazard Distance (ENOHD) beyond the aircraft’s altitude, posing risks to terrestrial observers. To resolve this conflict, an asymmetric parameterization strategy was developed for the two laser channels:

- Green Channel (Bathymetry): The PRR was maintained at 200 kHz to ensure high penetration in turbid waters. To compress the ENOHD to a safe limit of 527.83 m (below the 533.40 m flight altitude), the Field of View (FOV) was narrowed to 30°, concentrating the energy near the nadir. The laser beam divergence was maximized to 2.0 mrad to spatially average wave dynamics on the river surface, facilitating accurate beam refraction [21].
- IR Channel (Topography): To compensate for the narrow bathymetric FOV and provide a robust geometric frame for spectral analysis, the IR channel was set to an ultra-dense PRR of 900 kHz with a wider FOV of 41.19° and a sharp divergence of 0.3 mrad. This guaranteed precise delineation of the riparian zone even under aircraft roll conditions.

### 2.2. The Tri-Index Spectral Fusion Paradigm

Standard water extraction methodologies relying solely on a single index, such as the Normalized Difference Water Index (NDWI) [22], are systematically insufficient in complex ALB deployments. Riversides frequently transition into wetlands, and urban rivers are intersected by various gray infrastructure. HydroBound-ML integrates a Tri-Index Fusion model designed as a multi-layered filter:

1. Core Identification (NDWI): Highlights the fundamental water masses (both deep and shallow).
2. The Biological Shield (NDVI): The Normalized Difference Vegetation Index [23,24] is utilized to mathematically block the spatial “leakage” of the water mask into adjacent swamps, dense riparian vegetation, and flooded meadows.
3. The Abiotic Shield (NGRDI): The Normalized Green–Red Difference Index, calculated as (Green − Red)/(Green + Red), plays the most critical role in separating turbid water from structurally identical urban infrastructure (bridges, asphalt). A physical threshold limit is strictly enforced to decisively sever connections with gray infrastructure.

### 2.3. Contextual Machine Learning and the Multi-Scale Feature Space

A profound limitation of standard pixel-based classification is the inability to distinguish objects with identical radiometry but different physical textures. A pixel on the dark asphalt of a bridge may yield the exact same Tri-Index values as a pixel representing a deep, shadowed section of the river. The overall architecture developed to overcome this limitation is illustrated in Figure 3.

To overcome these spectral limitations, HydroBound-ML shifts to a high-dimensional spatial–spectral approach. The Random Forest (RF) classifier [14,25,26,27] does not evaluate isolated pixels. Instead, for every point, the algorithm dynamically calculates the spatial variance (standard deviation) and Gaussian Gradient Magnitudes (GGMs) across three distinct physical scales (Micro, Mid, and Macro, corresponding to user-defined metric radii). This expands the feature space to 14 dimensions, granting the model the contextual awareness to differentiate the smooth texture of a water body from the high-frequency roughness of a steel bridge truss. The specific selection and design of these 14 features were methodically driven by the physical properties of the fluvial environment. While flat pixel radiometry fails to separate turbid water from dark asphalt (due to the aforementioned “Information Ceiling”), their physical textures differ significantly. By capturing spatial variances and edge gradients at multiple discrete scales, the feature space is explicitly engineered to recognize the high-frequency surface roughness characteristic of civil infrastructure, contrasting it with the low-frequency, homogenous smoothness of water bodies.

Bootstrapping and the Human-in-the-Loop Protocol: To avoid the labor-intensive process of manually tracing rivers, the system employs an automated, bounded Otsu thresholding algorithm as a “Bootstrap” [28]. This generates an initial background dataset of the bulk water mass. However, purely automated Otsu methods suffer from “Background Dominance,” overwhelming any fine-tuning attempts. To counteract this, HydroBound-ML implements an Active Learning strategy known as the “Surgical Strike” protocol. When an analyst identifies a topological error (e.g., a mask bleeding over a bridge), their manual correction dynamically purges conflicting automated Otsu points within a 100 m radius. These high-weighted manual samples are subjected to Jitter Augmentation (radial spatial duplication and numerical feature noise cloning), forcing the RF decision trees to respect the newly defined boundary constraints without destroying the global river shape (Figure 4).

### 2.4. Semantic–Geometric Decoupling (Coarse-to-Fine OBIA)

During the algorithmic development, a fundamental phenomenon termed the “Pixel-based Texture Classification Edge Paradox” was identified. A machine learning model reliant on a sliding-window variance inherently cannot snap to a hard boundary (like a shoreline) without incurring a spatial penalty equal to half the window’s radius. If the window encompasses the shore, the high variance forces the model to classify the edge as non-water, physically pushing the predicted polygon away from the true shoreline.

To resolve this, HydroBound-ML decouples the semantic decision from the geometric execution:

- **Semantic ML (Coarse):** The RF model predicts the “meaning” of the landscape. It confidently identifies the core river while strictly avoiding bridges and asphalt, intentionally ignoring edge precision.
- **Geometric OBIA (Fine):** Simultaneously, the raw imagery is segmented into Simple Linear Iterative Clustering (SLIC) superpixels [29,30,31]. These superpixels generally adhere to and closely follow physical image gradients (e.g., natural shorelines).
- **Vectorized Fusion:** The coarse ML semantics are projected into the fine SLIC geometries. Using highly optimized hardware-accelerated operations (NumPy bincount) NumPy (version 2.4.6, available online: https://numpy.org, accessed on 27 August 2026), the system evaluates the ratio of “water” semantics within each superpixel in milliseconds. If a superpixel exceeds a defined ratio, its precise physical geometry is adopted into the final mask.

### 2.5. Big Data Operationalization and Software Architecture

The processing of Very High Resolution (VHR) datasets encompassing dozens of gigabytes traditionally paralyzes ssstandalone workstations, primarily due to I/O bottlenecks. HydroBound-ML mitigates this through a native Geographic Information System (GIS) architecture developed in Python (version 3.11.9; Python Software Foundation, Wilmington, DE, USA; available online: https://www.python.org, accessed on 27 August 2026). Instead of loading entire matrices into RAM, index generation is executed via the Rasterio Python library (version 1.4.4; available online: https://rasterio.readthedocs.io, accessed on 27 August 2026), which acts as an interface to GDAL’s (version 3.10.3; Open Source Geospatial Foundation; available online: https://gdal.org, accessed on 27 August 2026) Virtual Rasters (WarpedVRT), enabling sub-pixel alignment of disparate multi-sensor flight lines on the fly [32,33]. Processing is compartmentalized into a Macro-Chunking paradigm (blocks of 4096 × 4096 pixels).

Furthermore, the software resolves the “Small Fissle, Slow Render” conflict. All generated spectral indices are natively compiled as Cloud Optimized GeoTIFFs (COGs) with embedded internal image pyramids. This ensures that the interactive GUI (Figure 5) can fluidly render the workspace asynchronously, reducing RAM consumption by approximately 95% compared to standard float32 array loads.

It should be noted that during the software development phase, a large language model Gemini (version 3.1 Pro, Google LLC, Mountain View, CA, USA) was utilized strictly as an auxiliary programming and troubleshooting assistant. However, all core architectural decisions, hyperparameter tuning, and final pipeline integrations within the HydroBound-ML framework were independently verified, empirically tested, and finalized by the authors.

A comprehensive list of the core algorithmic hyperparameters utilized to achieve the reported results is provided in Table 1.

### 2.6. Hardware Environment and Computational Efficiency

The processing pipeline was executed on a high-performance virtualized workstation (VMware ESXi, version 8.0 Update 2; VMware, Inc., Palo Alto, CA, USA). The hardware configuration comprised an Intel^®^ Xeon^®^ Gold 6348 CPU @ 2.60 GHz (allocated 16 virtual cores) (Intel Corporation, Santa Clara, CA, USA), 256 GB of RAM, and an NVIDIA RTX A6000 (NVIDIA Corporation, Santa Clara, CA, USA) graphical processing unit with 48 GB of dedicated VRAM. Storage operations were distributed asymmetrically across a 2.0 TB SSD for active I/O processes and a 20.0 TB HDD for data archiving.

Under this configuration, the training of the Random Forest model—utilizing the highly augmented dataset and multi-scale textures—required approximately 1.5 h. Subsequent semantic prediction and geometric vectorization over the entire 20 km study area were completed in 25–30 min. This confirms the operational scalability of the developed Macro-Chunking and GDAL-accelerated architecture. This level of computational efficiency—processing dozens of gigabytes of VHR multi-spectral data in under 30 min on a single workstation node—demonstrates a critical operational advantage over heavy deep-learning architectures, which typically require multi-GPU clusters and prolonged inference times for large-scale spatial datasets of this magnitude.

### 2.7. Validation Strategy and Benchmark Methods

To rigorously evaluate the algorithmic performance and validate the necessity of the proposed hybrid approach, an intensive quantitative benchmark was designed. The model was trained using samples extracted from a representative 15 km stretch of the study area. Conversely, a spatially independent 5 km contiguous section—encompassing approximately 25% of the total dataset—was completely excluded from the model training phase and strictly reserved for final accuracy assessment. Establishing absolute ground truth for this validation segment alone required approximately 40 h of manual vectorization by an expert operator, highlighting the severe operational bottleneck this study aims to resolve.

This specific sub-area was deliberately selected due to its high morphological complexity and challenging topography. It encapsulates all critical elements that traditionally confound automated extraction algorithms: multiple intersecting civil engineering structures (bridges), densely vegetated ephemeral sandbars (fluvial islands), shallow shorelines, and numerous stones protruding above the water surface.

HydroBound-ML was benchmarked against five conventional delineation techniques utilizing area-based metrics (Precision, Recall, F1-Score, and Intersection over Union—IoU):

- **BDOT10k (OT_PTWP_A):** The official, state-maintained Polish National Topographic Database (Pol. Baza Danych Obiektów Topograficznych), compiled at a spatial detail level corresponding to a 1:10,000 scale map, representing a traditional static approach.
- **OTSU Thresholding:** A global, automated binarization technique applied to the NDWI matrix.

- **NDWI (≥0.1, ≥0.3, and ≥0.45):** Three distinct thresholding levels of the standard Normalized Difference Water Index.

While modern deep learning (DL) architectures (e.g., U-Net [15,34], DeepLabV3+ [35], SegFormer [36]) represent the state of the art in semantic segmentation, they were deliberately excluded from this comparative baseline. In the specific context of operational ALB processing, DL models require massive, densely annotated training datasets (which are unavailable for this specific sensor configuration) and introduce prohibitive computational and memory overhead when inferring across 4096 × 4096 VHR tiles without downsampling. Furthermore, the necessity for a ‘Human-in-the-Loop’ architecture demands near real-time model retraining (within minutes) on standard workstation hardware, a constraint that currently precludes heavy DL architectures. Therefore, the benchmarking focuses on evaluating the proposed hybrid framework against standard, computationally accessible indices and operational GIS databases utilized in everyday hydrographic practice. In the present study, Random Forest represents the conventional supervised machine-learning component of the proposed framework; the methods not included in the comparison are primarily deep-learning (DL) segmentation architectures.

## 3. Results

The application of HydroBound-ML to the complex fluvial dataset of the Vistula and Pilica rivers demonstrated exceptional robustness against civil infrastructure noise. The model successfully decoupled the river topology from overlapping railway bridges and densely vegetated ephemeral islands.

### 3.1. Internal Machine Learning Performance

The internal performance of the trained Random Forest classifier was initially evaluated to ensure baseline convergence. To rigorously prevent spatial data leakage during this phase, an initial pool of 176,598 manually annotated, unaugmented sample points was first grouped using a density-based spatial clustering algorithm (DBSCAN [37]) to identify spatially contiguous click-clusters. A Group Shuffle Split was then employed to partition these clusters into training and internal validation subsets at an 80:20 ratio, guaranteeing that spatially adjacent points from a single manual interaction strictly fell into the same subset. Crucially, spatial Jitter Augmentation (×20) was subsequently applied exclusively to the training subset to improve model generalization against radiometric noise. Evaluated on the strictly held-out, non-augmented validation set, the model achieved a realistic and robust global Precision of 0.91, a Recall of 0.94, an F1-score of 0.92, and an Intersection over Union (IoU) of 0.86. Consequently, the definitive assessment of the framework’s true reliability and generalization capability was fundamentally based on the spatially independent external evaluation (detailed in Section 3.2).

### 3.2. External Validation and Benchmarking

The quantitative external evaluation against the manually verified ground truth polygon demonstrates the limitations of standard remote sensing indices when applied to complex fluvial environments. It is important to emphasize that the performance metrics reported in Table 2 for HydroBound-ML reflect the purely automated pipeline (Semantic ML + Geometric OBIA fusion), prior to any manual refinement via the Expert Override module. This ensures a strictly fair, unassisted comparison against the fully automated baseline methods. The results of the comparative analysis are detailed in Table 2 and visually summarized in Figure 6.

In addition to area-based metrics, the spatial fidelity of the predicted boundaries was evaluated. As detailed in the Discussion (Section 4), the application of the Micro scale parameter inherently dictates a mathematically predictable spatial retreat from the absolute physical shoreline. For the HydroBound-ML framework, the resulting boundary displacement associated with the Micro-scale contextual window was approximately 1.5 m.

In stark contrast, conventional thresholding techniques (OTSU and NDWI ≥ 0.1) exhibited severe infrastructural spill-over, resulting in boundary deviations frequently exceeding tens of meters around bridges and densely vegetated riparian zones. Furthermore, a McNemar’s statistical test confirmed that the classification performance differences between HydroBound-ML and all baseline methods were statistically significant (*p* < 0.001).

When compared to standard extraction techniques, the superiority of the hybrid ML-OBIA approach becomes evident. Conventional index-based methods, such as the NDWI (threshold ≥ 0.1) and global OTSU thresholding, systematically failed to isolate the water mass from adjacent infrastructure. In these cases, the predicted water polygons “spilled over” the riverbanks, bleeding heavily onto bridges, asphalt roads, and adjacent terrain due to spectral similarities in the optical and NIR spectrums. This phenomenon directly translates to their poor Precision scores (0.49 for NDWI ≥ 0.1 and 0.55 for OTSU). While enforcing stricter sensitivity (NDWI ≥ 0.45) curtailed false positives, it significantly reduced the Recall (0.02), rendering the mask practically useless.

State-maintained topographic databases such as BDOT10k achieved a high Recall (0.99), but their Precision was considerably lower (0.76). This suggests that, although the database covered most of the water area, its geometry did not always correspond to the actual river boundary at the time of the ALB survey. This is particularly relevant in fluvial environments, where erosion and deposition can cause relatively rapid changes in the river channel. The proposed method showed the opposite pattern, with a Precision of 0.99 and a Recall of 0.91. By relying on spatial multi-scale variance rather than flat pixel radiometry, the model consistently recognized bridges and protruding rocks as non-water entities, entirely preventing the “spill-over” effect. It therefore produced very few false-positive water classifications, although some water areas were not detected. For ALB preprocessing, this trade-off is useful because the inclusion of non-water areas may affect the subsequent waveform decomposition.

### 3.3. Topological Integrity and Expert Override

A critical finding was the topological necessity of Spatial Boolean Set Theory. Initial attempts to manually refine mask “leakages” or gaps using zero-area polylines (zippering) resulted in severe geometric failures during export. To address this, a dedicated “Expert Override” module was implemented, allowing for hardware-accelerated Boolean topological editing (Union/Difference) secured by self-healing vector geometry. This framework provides a robust, error-free method to refine the mask at critical sites—ranging from excising structural pillars of bridges (Figure 7) to filling ephemeral data voids in narrow channel bifurcations or manually delineating the boundary in complex, narrow passes where automated segmentation reached its morphological limit. Consequently, this topological refinement approach resulted in a substantial reduction in recall errors during subsequent Exponential Decomposition processes.

Beyond infrastructural challenges, the algorithm’s geometric fidelity was rigorously evaluated against highly irregular natural shorelines. As illustrated in Figure 8, the integration of SLIC superpixels enables the vector mask to meticulously adhere to complex morphological features, including ephemeral sandbars, sharp meanders, and bifurcating channels. The algorithm natively resolves complex spatial topologies, automatically generating multi-part polygons with valid inner rings (holes) for riverine islands without requiring manual digitization.

## 4. Discussion

The integration of multi-scale variance naturally introduces a minor, mathematically predictable spatial retreat from the absolute physical shoreline. In the context of ALB Exponential Decomposition, this conservative buffer is actively beneficial. It acts as an operational safety margin, guaranteeing absolute avoidance of steep terrestrial banks that could otherwise corrupt the bathymetric point cloud with high-intensity land returns. Because the geometry is driven by the OBIA SLIC superpixels rather than the blurry ML variance window, this buffer remains sharp and topographically consistent. In practice, the average magnitude of this spatial retreat for the analyzed dataset is approximately 1.5 m. This value is not arbitrary; it is a direct mathematical consequence of the base perception window (Scale 1: Micro) defined during the spatial feature extraction phase and the subsequent morphological smoothing parameters applied to the raw predictions.

Beyond ALB processing, the open architecture and lack of dependency on proprietary LiDAR software enable HydroBound-ML to serve as a standalone framework for hydrographic vectorization. The capability to export DTM-attributed 3D polygon grids allows the rapid updating of national spatial databases (e.g., BDOT10k) and serves as critical input for flood modeling.

### Limitations of the Methodology 

Despite its robust performance, the proposed methodology is subject to specific operational limitations. Primarily, the algorithm’s efficacy is fundamentally dependent on the simultaneous acquisition of high-resolution, multi-spectral (RGB and NIR) imagery alongside the ALB mission. Operations conducted under severe lighting conditions, extreme sun glint, or campaigns relying solely on the LiDAR point cloud intensity without supplementary optical data cannot fully exploit the Tri-Index Spectral Fusion paradigm. Additionally, a fundamental limitation of the current study is its reliance on a single, albeit morphologically complex, geographic site (a 20 km stretch of the Vistula and Pilica rivers). While the spatially independent test section ensures rigorous local validation, it cannot fully demonstrate the algorithm’s generalization capabilities across drastically different environmental conditions, such as varying water turbidity levels, differing riparian vegetation species, or distinct coastal morphologies. Future research must involve multi-site validation across diverse climatic zones and alternative sensor configurations to definitively establish the framework’s global robustness.

Beyond data acquisition constraints, paradoxical morphological edge cases inevitably present distinct challenges for the automated pipeline, as visually demonstrated in Figure 9. First, while the inherent mathematical property of the multi-scale variance window introduces a conservative shoreline buffer (approximately 1.5 m, shown in Figure 9A) that is highly beneficial for preventing false target extraction in ALB, it inherently truncates extremely shallow, near-shore water margins. Furthermore, because the semantic classification is strictly driven by 2D optical orthomosaics, the algorithm delineates the visible water extent. Consequently, the vectorized boundary inherently follows objects occluding the physical shoreline from above, such as overhanging tree canopies (also observable in Figure 9A). Second, topological fracturing can occur within exceptionally narrow, bifurcating fluvial channels (Figure 9B). When the channel width falls below the structural resolution of the SLIC superpixel segmentation (driven by the Scale 1 parameter), the algorithm may break the vector continuum, falsely classifying these extremely narrow straits as land bridges.

These specific artifacts necessitate the application of the hardware-accelerated Boolean editing tool (Expert Override) to manually restore the topological network prior to the ALB export. Finally, although the geometric execution is heavily optimized, the initial parameter tuning and Random Forest training phase still require manual intervention and an understanding of spatial feature engineering, precluding a fully autonomous, “one-click” deployment in unstructured environments.

## 5. Conclusions

The results demonstrate that HydroBound-ML provides an accurate, scalable, and reproducible approach for automated water-boundary delineation in large airborne bathymetric LiDAR datasets. By establishing a multi-dimensional feature space and acknowledging the “Edge Paradox”, the tool effectively mitigates spectral confusion over civil infrastructure. As demonstrated by comprehensive benchmark testing, HydroBound-ML significantly outperforms conventional methods (NDWI, OTSU, static databases) by decoupling semantic classification from geometric delineation, achieving an F1-Score of 0.95 and virtually eliminating false-positive “spill-overs” into adjacent land masses (Precision = 0.99). Coupled with advanced Big Data handling techniques—VRT Macro-Chunking, COG pyramiding, and Boolean vectorization—the software provides a robust, scalable, and highly precise image-based preprocessing tool for generating the water-surface selector required by downstream Airborne Lidar Bathymetry processing.

## Figures and Tables

**Figure 1:**
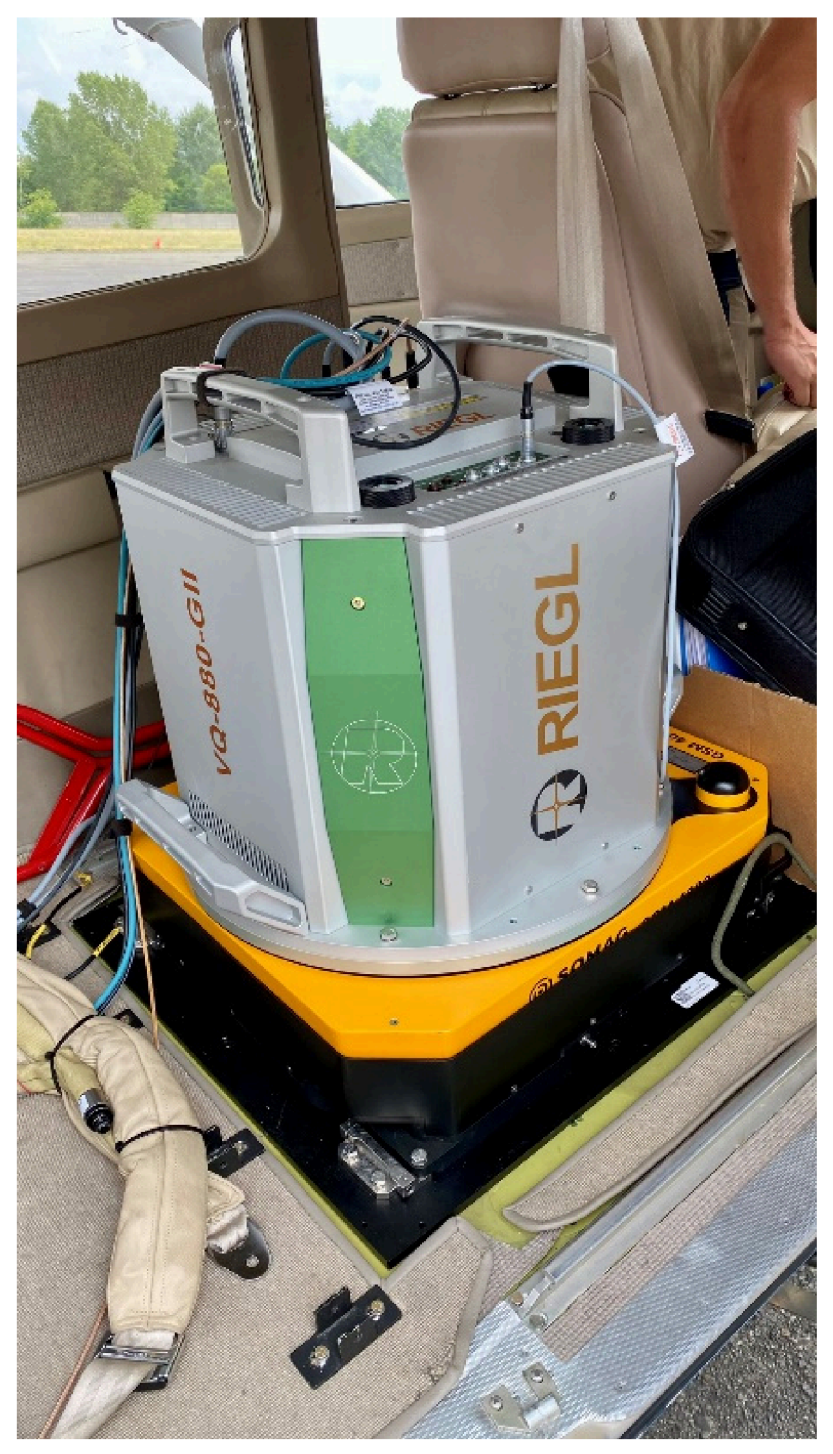
The topo-bathymetric system Riegl VQ-880-GII mounted inside a Cessna T206H (Textron Aviation Inc., Wichita, KS, USA) aircraft.

**Figure 2:**
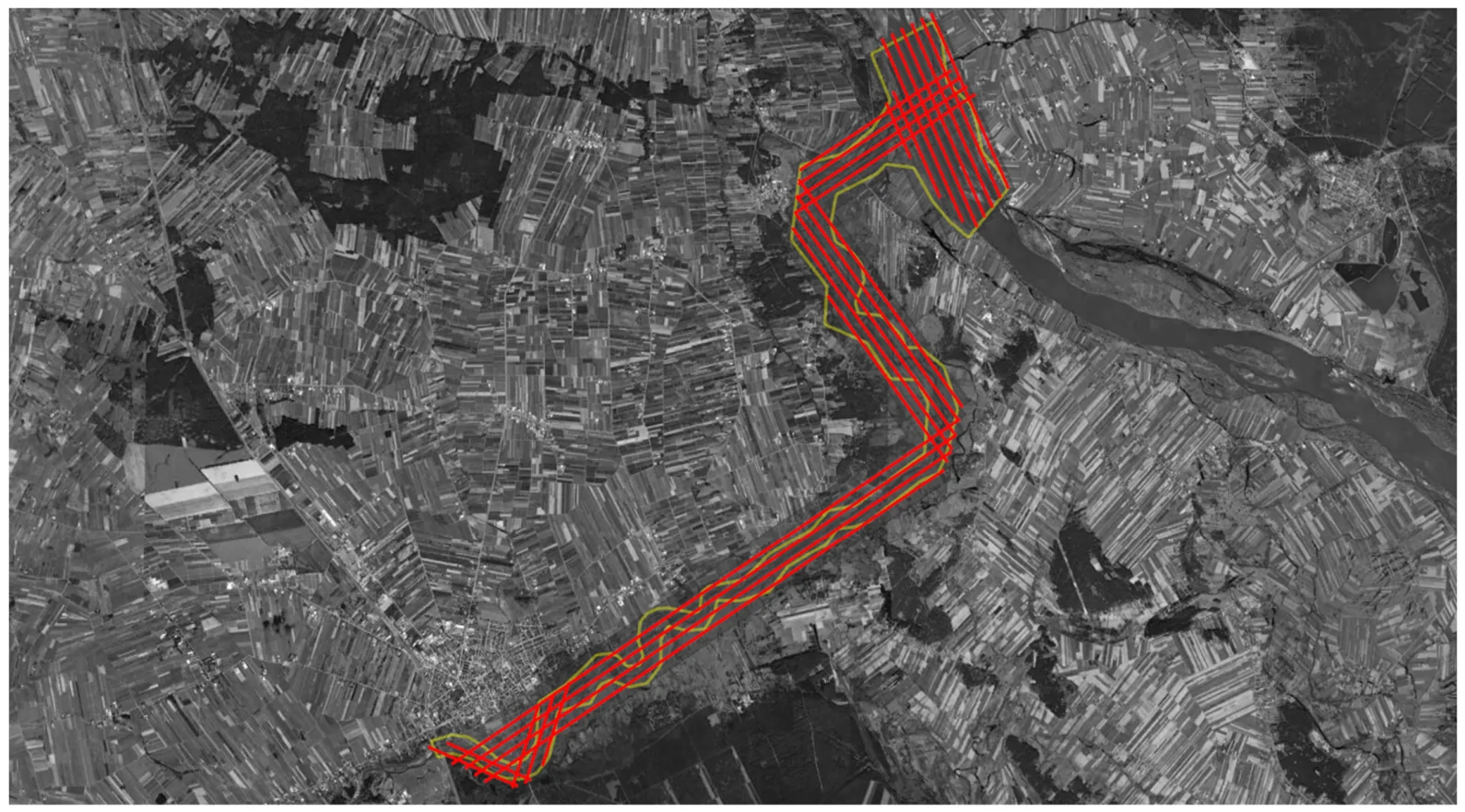
Orthographic view of the flight plan over the Pilica and Vistula rivers, illustrating the 50–60% transverse overlap (sidelap) utilized to mitigate sun glint and ensure redundant point density. The red lines indicate the flight lines, and the yellow area represents the surveyed region.

**Figure 3:**
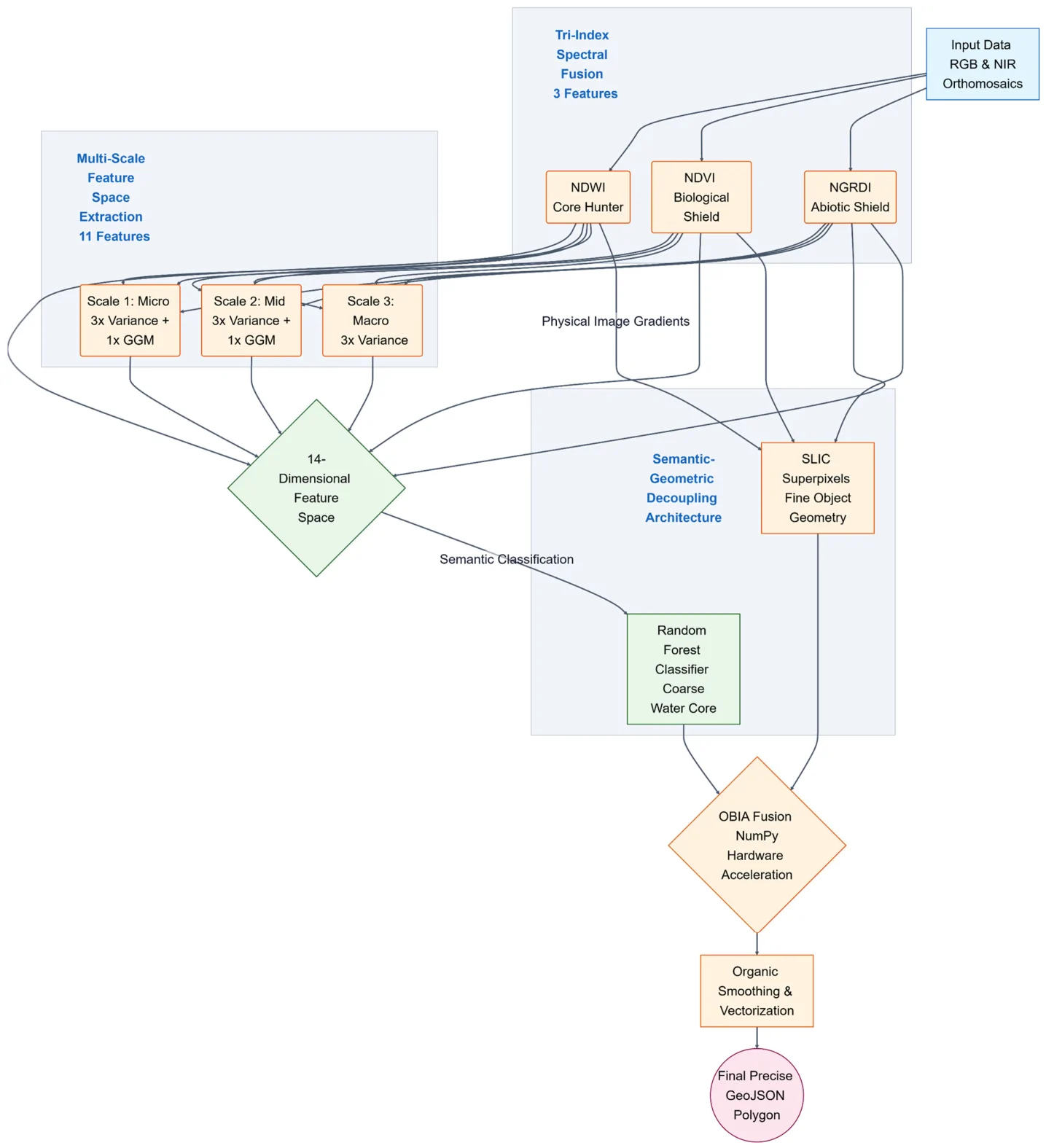
Diagram of the 14-dimensional multi-scale feature space architecture.

**Figure 4:**
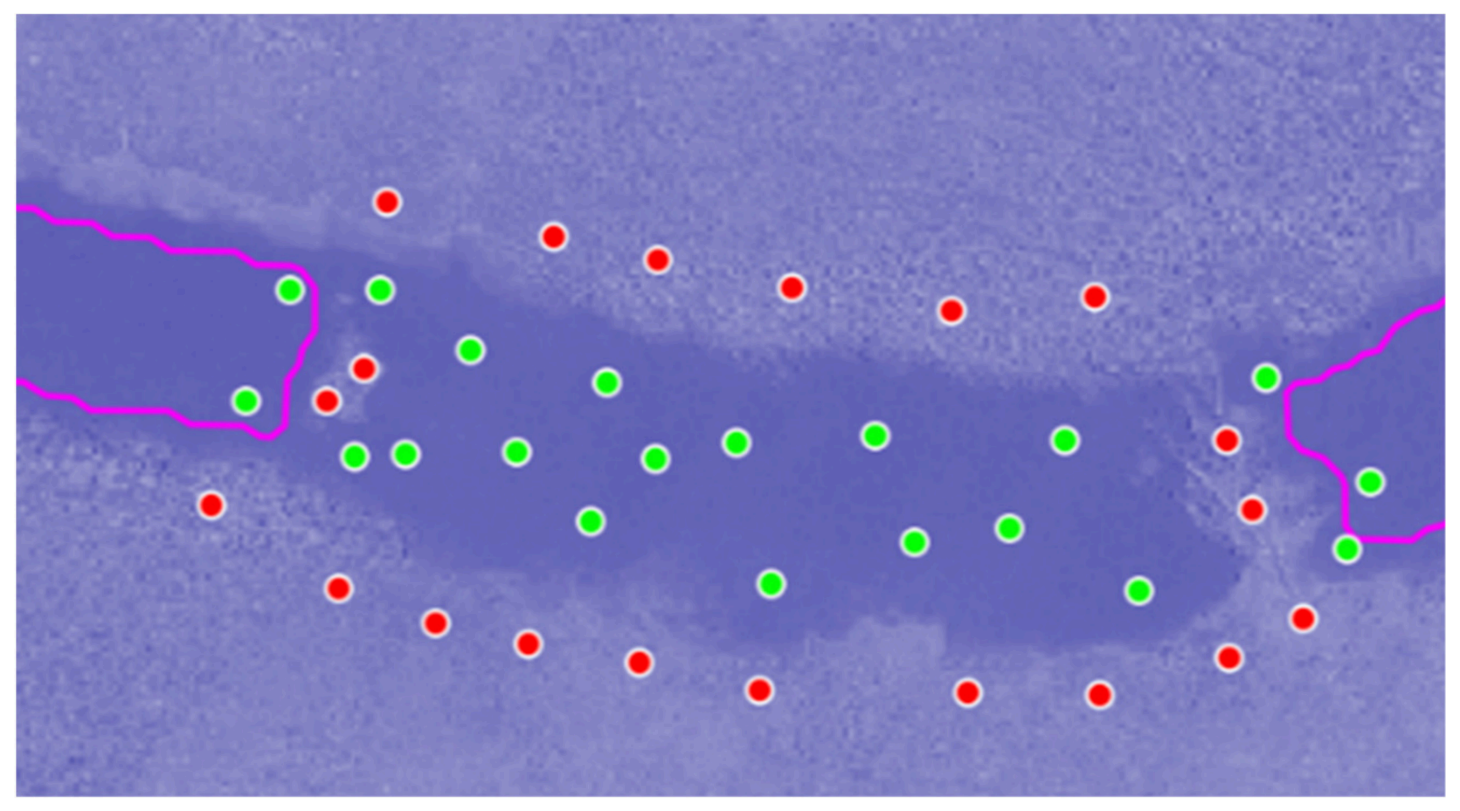
Interactive machine learning interface with an NDWI raster background. The purple lines represent the water boundary polygon from the previous iteration, while the user-provided manual samples (green dots for water, red dots for non-water) are selected during the model retraining phase to surgically correct the algorithm’s predictions via the Human-in-the-Loop Protocol.

**Figure 5:**
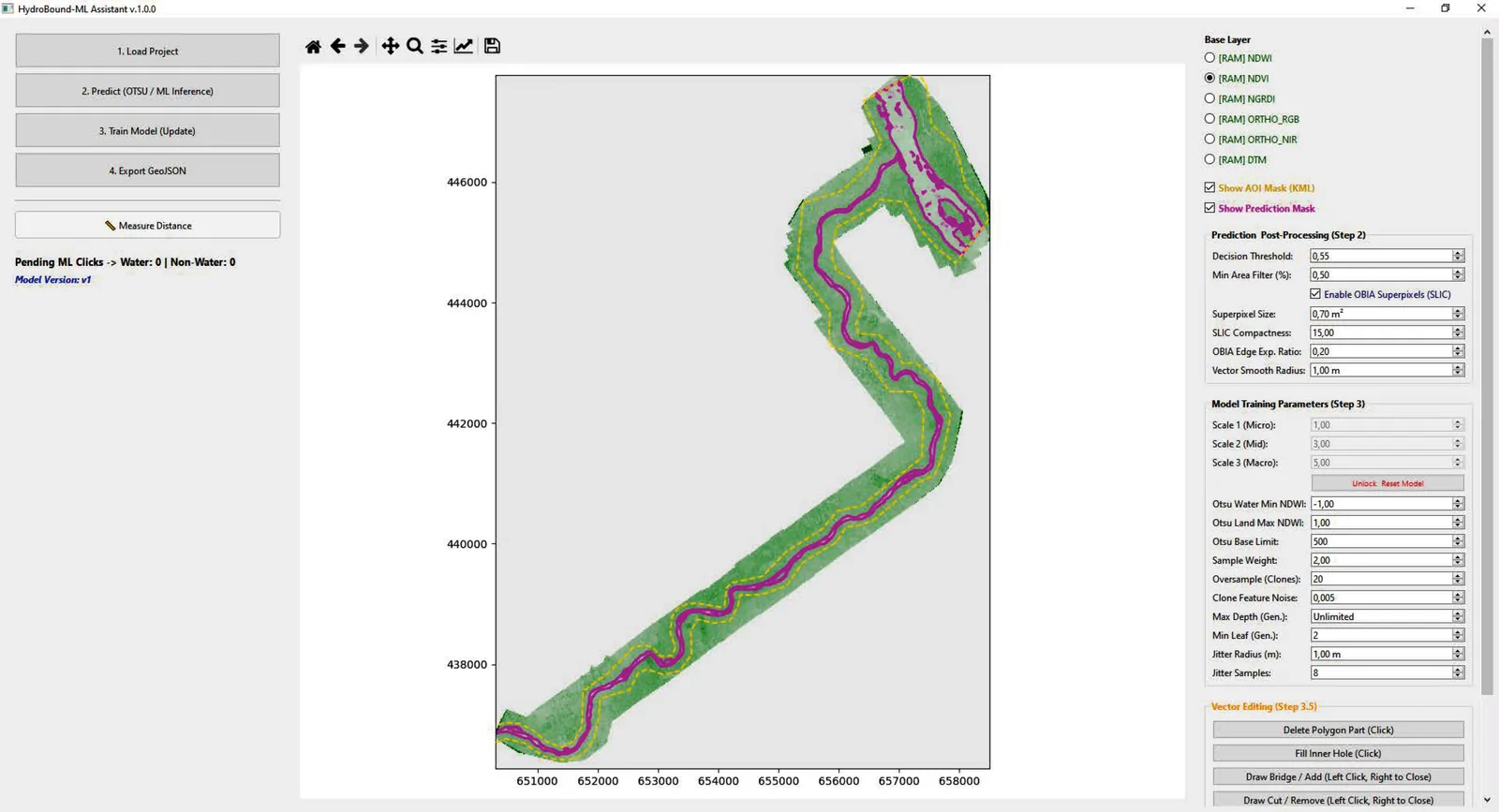
The HydroBound-ML Assistant graphical user interface, displaying the generated precise vector mask overlaid on the calculated spectral indices, alongside the parameter control panel. The green-toned background represents the NDVI raster, while the purple lines indicate the water boundary vector generated by the HydroBound-ML software.

**Figure 6:**
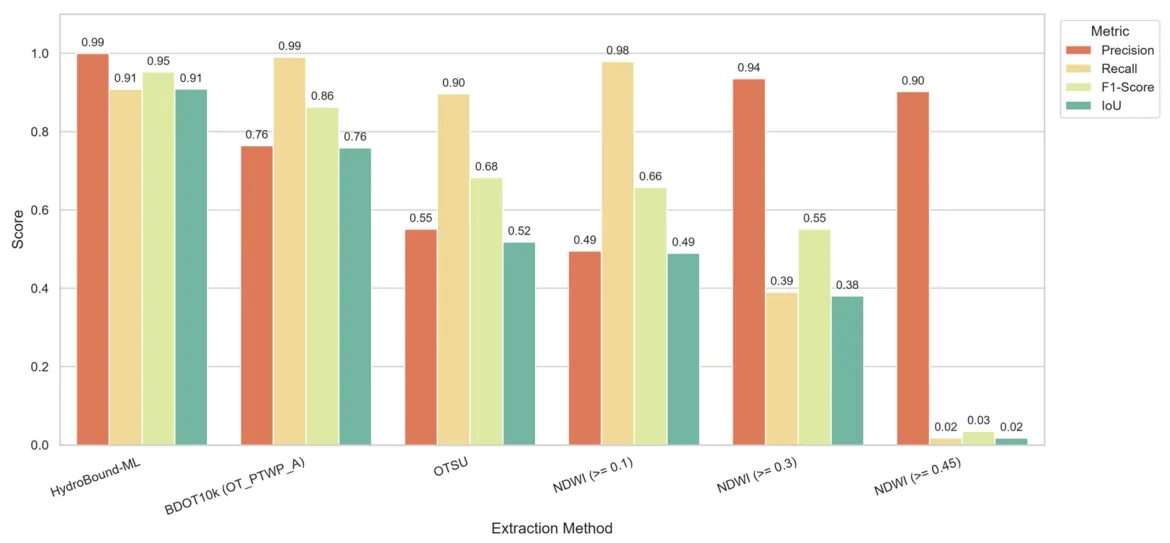
Performance metrics of water delineation methods.

**Figure 7:**
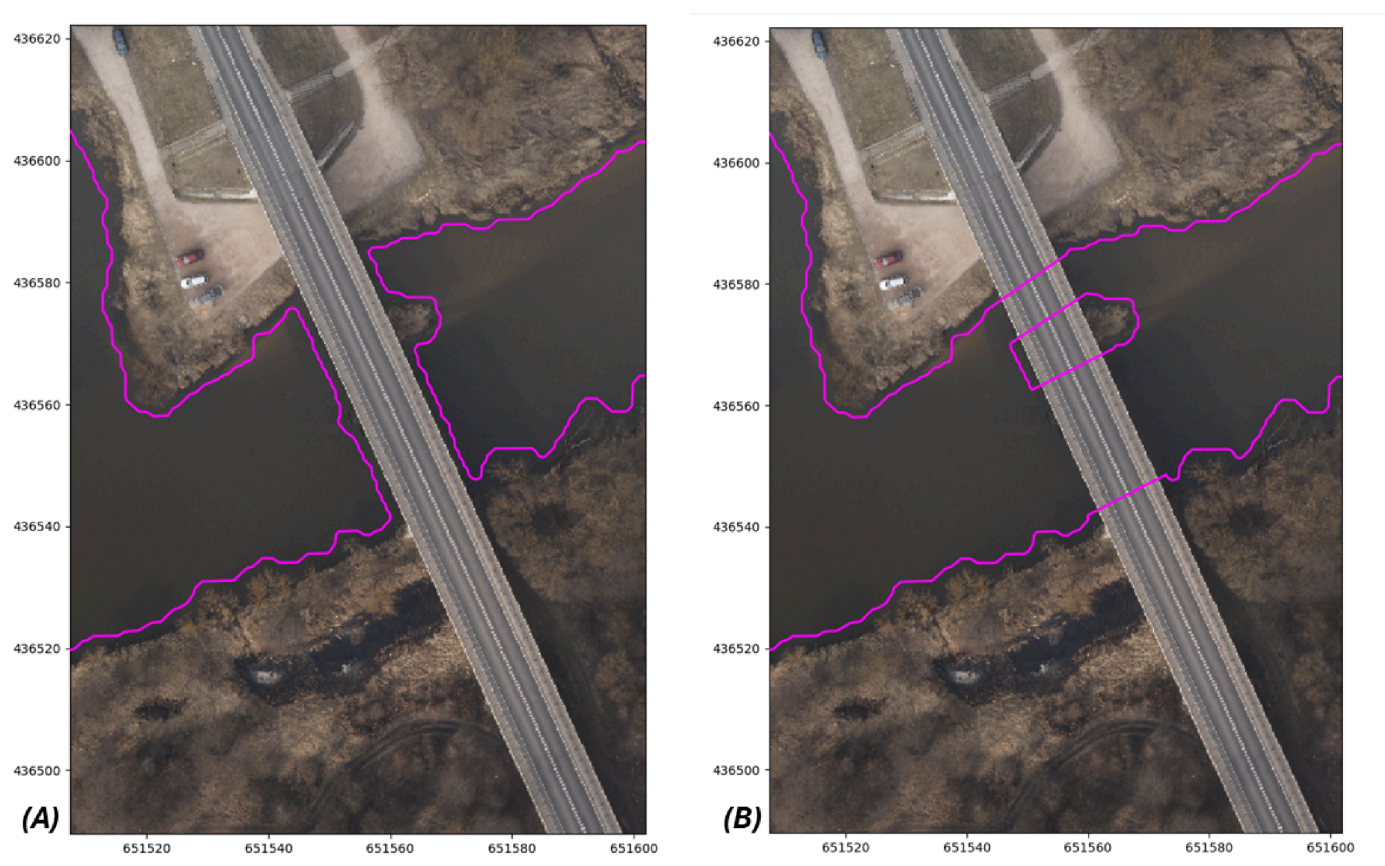
(**A**) A raw ortho-image showing a bridge intersecting a river alongside the algorithm’s initial mask. (**B**) The application of the Expert Override Boolean editing tool to topologically refine the mask at the bridge pillars, facilitating bathymetric target extraction from the water beneath the bridge. The purple lines represent the vector boundary generated by the HydroBound-ML algorithm, overlaid on an RGB orthophotomap background.

**Figure 8:**
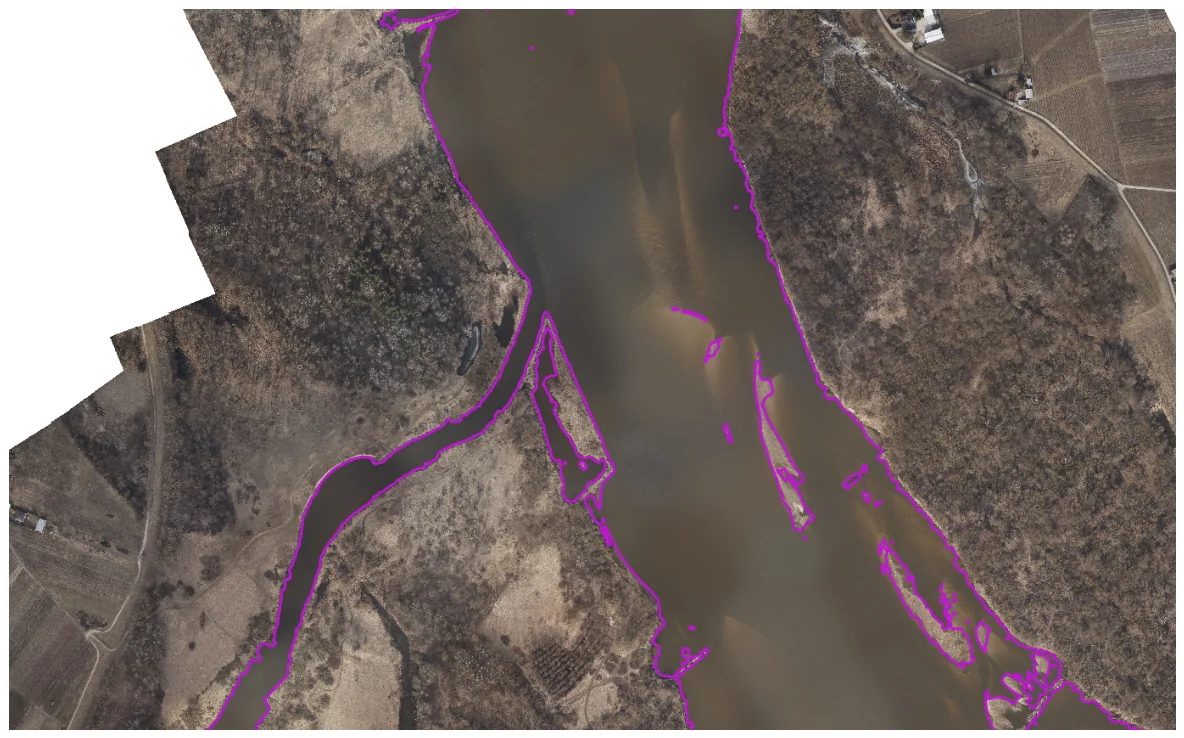
Demonstration of topological correctness and high geometric fidelity. The algorithm successfully delineates complex natural features, including river bifurcations and internal islands, maintaining sharp physical boundaries driven by the OBIA SLIC superpixels. The purple lines indicate the vector boundary generated by the HydroBound-ML algorithm, overlaid on an RGB orthophotomap raster background.

**Figure 9:**
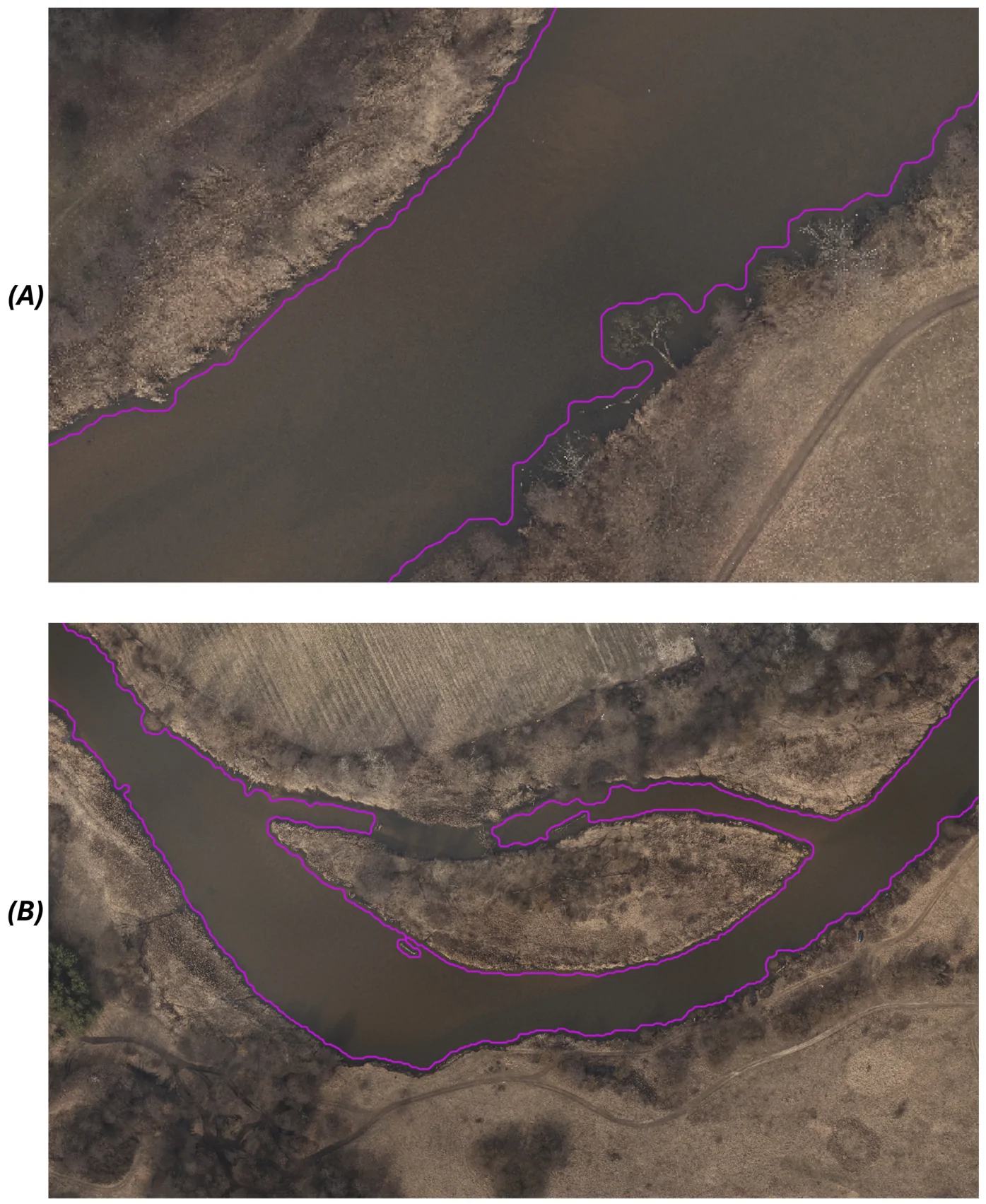
Operational limitations and morphological edge cases of the automated extraction. (**A**) The mathematically predictable spatial buffer (approx. 1.5 m) retreating from the physical shoreline, alongside the boundary tracing overhanging tree canopies due to optical occlusion. (**B**) Topological fracturing in an extremely narrow river branch caused by the spatial resolution limits of the superpixel segmentation. The purple lines indicate the vector boundary generated by the HydroBound-ML algorithm, overlaid on an RGB orthophotomap raster background.

**Table 1:** Comprehensive list of key hyperparameters utilized in the HydroBound-ML framework.

Component	Parameter	Default Value
Feature Space (Scales)	Scale 1: Micro Radius	1.00 m
Feature Space (Scales)	Scale 2: Mid Radius	3.00 m
Feature Space (Scales)	Scale 3: Macro Radius	5.00 m
Random Forest (ML)	Number of Estimators (Trees)	150
Random Forest (ML)	Max Depth	Unlimited
Random Forest (ML)	Min Samples Leaf	2
Random Forest (ML)	Sample Weight	2.00
OBIA (SLIC)	Superpixel Size	0.70 m^2^
OBIA (SLIC)	Compactness	15.00
OBIA Fusion	ML Decision Threshold (Probability)	0.55
OBIA Fusion	OBIA Edge Exp. Ratio	0.20
OBIA Fusion	Min Area Filter	0.50%
OBIA Fusion	Vector Smooth Radius	1.00 m
Human-in-the-Loop	Active Purge Radius	100.0 m
Augmentation	Jitter Radius	1.00 m
Augmentation	Jitter Samples	8
Augmentation	Oversample (Clones)	20
Augmentation	Clone Feature Noise	0.005

**Table 2:** Area-based performance metrics of evaluated water delineation methods.

Extraction Method	Precision	Recall	F1-Score	IoU
HydroBound-ML	0.99	0.91	0.95	0.91
BDOT10k (OT_PTWP_A)	0.76	0.99	0.86	0.76
OTSU	0.55	0.90	0.68	0.52
NDWI (≥0.1)	0.49	0.98	0.66	0.49
NDWI (≥0.3)	0.94	0.39	0.55	0.38
NDWI (≥0.45)	0.90	0.02	0.03	0.02

## Data Availability

The HydroBound-ML Assistant (v.1.0.7) is available as an open-source project under the MIT License. The repository contains the complete source code, documentation, and user guides to ensure full transparency and reproducibility of the workflow: https://github.com/PWroblewskiWAT/HydroBound-ML (accessed on 21 September 2026). The original data presented in the study, including the training dataset (Magnuszew_2026), the pre-trained machine learning model, corresponding performance metrics, full project structure, and auxiliary validation data, are openly available in FigShare at https://doi.org/10.6084/m9.figshare.33366177 (accessed on 21 September 2026).

## Abbreviations

The following abbreviations are used in this manuscript:

ALB	Airborne Lidar Bathymetry
BDOT10k	Polish National Topographic Database (polish: Baza Danych Obiektów Topograficznych) at a scale of 1:10,000
COG	Cloud Optimized GeoTIFF
CPU	Central Processing Unit
DTM	Digital Terrain Model
ENOHD	Extended Nominal Ocular Hazard Distance
FOV	Field of View
GDAL	Geospatial Data Abstraction Library
GenAI	Generative Artificial Intelligence
GGMs	Gaussian Gradient Magnitudes
GIS	Geographic Information System
GPU	Graphical Processing Unit
GUI	Graphical User Interface
HDD	Hard Disk Drive
IoU	Intersection over Union
IR	Infrared
ML	Machine Learning
NDVI	Normalized Difference Vegetation Index
NDWI	Normalized Difference Water Index
NGRDI	Normalized Green–Red Difference Index
NIR	Near-Infrared
OBIA	Object-Based Image Analysis
PRR	Pulse Repetition Rate
RAM	Random Access Memory
RF	Random Forest
RGB	Red, Green, Blue
SLIC	Simple Linear Iterative Clustering
SSD	Solid State Drive
VHR	Very High Resolution
VRAM	Video Random Access Memory
VRT	Virtual Raster

## References

[1] Awadallah M.O.M., Juárez A., Alfredsen K. (2022). Comparison between Topographic and Bathymetric LiDAR Terrain Models in Flood Inundation Estimations. Remote Sens..

[2] Szafarczyk A., Toś C. (2023). The Use of Green Laser in LiDAR Bathymetry: State of the Art and Recent Advancements. Sensors.

[3] Xing S., Wang D., Xu Q., Lin Y., Li P., Jiao L., Zhang X., Liu C. (2019). A Depth-Adaptive Waveform Decomposition Method for Airborne LiDAR Bathymetry. Sensors.

[4] Mandlburger G., Hauer C., Wieser M., Pfeifer N. (2015). Topo-Bathymetric LiDAR for Monitoring River Morphodynamics and Instream Habitats—A Case Study at the Pielach River. Remote Sens..

[5] Fernandez-Diaz J.C., Carter W.E., Shrestha R.L., Glennie C.L. (2014). Now You See It… Now You Don’t: Understanding Airborne Mapping LiDAR Collection and Data Product Generation for Archaeological Research in Mesoamerica. Remote Sens..

[6] Pfennigbauer M., Ullrich A., Steinbacher D., Aufleger M. (2011). High-Resolution Hydrographic Airborne Laser Scanner for Surveying Inland Waters and Shallow Coastal Zones. Proceedings of the SPIE—The International Society for Optical Engineering.

[7] Wang D., Xing S., He Y., Yu J., Xu Q., Li P. (2022). Evaluation of a New Lightweight UAV-Borne Topo-Bathymetric LiDAR for Shallow Water Bathymetry and Object Detection. Sensors.

[8] Tao B., Li J., Guo W., He Y., Li Y., Huang H., Yu J., Mao Z. (2022). Precise Detection of Water Surface through the Analysis of a Single Green Waveform from Bathymetry LiDAR. Opt. Express.

[9] Li N., Truong M.-L., Schwarz R., Pfennigbauer M., Ullrich A. (2024). Deep Learning Assisted Exponential Waveform Decomposition for Bathymetric LiDAR. The International Archives of the Photogrammetry, Remote Sensing and Spatial Information Sciences.

[10] Kim H., Jung M., Lee J., Wie G. (2023). Progressive Gaussian Decomposition of Airborne Bathymetric LiDAR Waveform for Improving Seafloor Point Extraction. Appl. Sci..

[11] Kim H., Lee J. (2025). Waveform Analysis for Enhancing Airborne LiDAR Bathymetry in Turbid and Shallow Tidal Flats of the Korean West Coast. Remote Sens..

[12] Frazier P.S., Page K.J. (2000). Water Body Detection and Delineation with Landsat TM Data. Photogramm. Eng. Remote Sens..

[13] Chen Y., Fan R., Yang X., Wang J., Latif A. (2018). Extraction of Urban Water Bodies from High-Resolution Remote-Sensing Imagery Using Deep Learning. Water.

[14] Jiang Z., Wen Y., Zhang G., Wu X. (2022). Water Information Extraction Based on Multi-Model RF Algorithm and Sentinel-2 Image Data. Sustainability.

[15] Liu Y., Xie Y., Rui X., Zhang H., Ge J. (2026). A Multi-Scale Attention U-Net for Water Body Extraction Under Urban Shadow Interference. Water.

[16] Muhammad U., Hoque M.Z., Wang W., Oussalah M. (2022). Patch-Based Discriminative Learning for Remote Sensing Scene Classification. Remote Sens..

[17] Zhai M., Shen H., Cao Q., Ding X., Xin M. (2025). Water Body Extraction Methods for SAR Images Fusing Sentinel-1 Dual-Polarized Water Index and Random Forest. Sensors.

[18] Smeeckaert J., Mallet C., David N., Chehata N., Ferraz A. (2013). Large-Scale Classification of Water Areas Using Airborne Topographic LiDAR Data. Remote Sens. Environ..

[19] Song A. (2025). TAGNet: A Tidal Flat-Attentive Graph Network Designed for Airborne Bathymetric LiDAR Point Cloud Classification. ISPRS Int. J. Geo-Inf..

[20] (2014). Safety of Laser Products—Part 1: Equipment Classification and Requirements.

[21] Birkebak M., Eren F., Pe’eri S., Weston N. (2018). The Effect of Surface Waves on Airborne Lidar Bathymetry (ALB) Measurement Uncertainties. Remote Sens..

[22] McFeeters S.K. (1996). The Use of the Normalized Difference Water Index (NDWI) in the Delineation of Open Water Features. Int. J. Remote Sens..

[23] Rouse J., Haas R.H., Schell J.A., Deering D. (1973). Monitoring Vegetation Systems in the Great Plains with ERTS.

[24] Tucker C.J. (1979). Red and Photographic Infrared Linear Combinations for Monitoring Vegetation. Remote Sens. Environ..

[25] Belgiu M., Drăguţ L. (2016). Random Forest in Remote Sensing: A Review of Applications and Future Directions. ISPRS J. Photogramm. Remote Sens..

[26] Breiman L. (2001). Random Forests. Mach. Learn..

[27] Zhou Y., Zhang Y., Zhu G., Shen C., Tian Y., Zhou J., Guo Y., Hu J., Qiu G. (2026). Enhanced Machine Learning for Reliable Water Body Extraction of Plateau Wetlands Caohai Using Remote Sensing and Big Geospatial Data from Optical Zhuhai-1 and Radar Sat-2 Satellites. Land.

[28] Otsu N. (1979). A Threshold Selection Method from Gray-Level Histograms. IEEE Trans. Syst. Man. Cybern..

[29] Achanta R., Shaji A., Smith K., Lucchi A., Fua P., Süsstrunk S. (2012). SLIC Superpixels Compared to State-of-the-Art Superpixel Methods. IEEE Trans. Pattern Anal. Mach. Intell..

[30] Blaschke T. (2010). Object Based Image Analysis for Remote Sensing. ISPRS J. Photogramm. Remote Sens..

[31] Hafizi H., Kalkan K. (2020). Evaluation of Object-Based Water Body Extraction Approaches Using Landsat-8 Imagery. J. Aeronaut. Space Technol..

[32] Billson J., Islam M.S., Sun X., Cheng I. (2023). Water Body Extraction from Sentinel-2 Imagery with Deep Convolutional Networks and Pixelwise Category Transplantation. Remote Sens..

[33] Gharbia R. (2023). Deep Learning for Automatic Extraction of Water Bodies Using Satellite Imagery. J. Indian Soc. Remote Sens..

[34] Ronneberger O., Fischer P., Brox T. (2015). U-Net: Convolutional Networks for Biomedical Image Segmentation. arXiv.

[35] Chen L.-C., Zhu Y., Papandreou G., Schroff F., Adam H. (2018). Encoder-Decoder with Atrous Separable Convolution for Semantic Image Segmentation. arXiv.

[36] Xie E., Wang W., Yu Z., Anandkumar A., Alvarez J.M., Luo P. (2021). SegFormer: Simple and Efficient Design for Semantic Segmentation with Transformers. arXiv.

[37] Ester M., Kriegel H., Sander J., Xu X. (1996). A Density-Based Algorithm for Discovering Clusters in Large Spatial Databases with Noise. KDD-96 Proceedings.

